# Qualitative exploration of how clinicians navigate divergence in older adult and family caregiver priorities

**DOI:** 10.1186/s12877-025-06762-3

**Published:** 2025-12-17

**Authors:** Martina Azar, Katherine A. Kennedy, Marla L. Clayman, Emma D. Quach

**Affiliations:** 1 New England Geriatrics Research Education Clinical Center, 150 S. Huntington Ave, Jamaica Plain, Huntington, MA 02130 USA; 2 Transformative Health Systems Research to Improve Veteran Equity and Independence (THRIVE) Center of Innovation, VA Providence Healthcare System, Providence, RI, USA, 830 Chalkstone Avenue, Providence, RI 02908 USA; 3 Center for Healthcare Optimization and Implementation Research, Bedford, VA Bedford; 200 Springs Road, MA 01730 USA; 4 New England Geriatrics Research Education Clinical Center, VA Bedford, 200 Springs Road, Bedford, MA 01730 USA; 5 University of Massachusetts Chan Medical School, 55 N. Lake Ave, Worcester, MA 01655 USA; 6 Brown University Health, Rhode Island Hospital, 1 Hoppin St, Providence, RI 02903 USA

**Keywords:** Priorities, Communication, Qualitative analysis, Veterans, Age-friendly environment

## Abstract

Clinicians often elicit and incorporate patient priorities into care plans. This process is complicated when patients and their family caregivers express incongruent priorities. We investigated patient and family caregiver priorities expressed during medical visits and clinician responses to divergent priorities. Methods: Our data consisted of four first-time medical appointments, each attended by an older patient, at least one family caregiver, and a team of geriatrics clinicians. We audio-recorded and transcribed the appointments, each lasting at least 120 min, and performed thematic analysis of the expressed priorities and clinician responses. Results: When incongruence in priorities emerged, clinicians responded with acknowledgement to each party’s priority and highlighted the incongruence itself, pointing out common ground when present, and pivoted to care planning, while exhibiting warmth and avoiding taking sides. Conclusions: Medical visits with patients and their family caregivers with divergent views present an opportunity for clinicians to promote candor and perspective-taking while finding common ground and assist throughout complex conversations. Further research should explore divergent priorities in larger patient samples with more diversity in cognitive ability and in varied healthcare settings.

## Introduction

In the U.S., older adults with physical and mental health comorbidities often rely on family caregivers to meet their daily physical, social, and mental health needs. In 2022, 34.2 million family caregivers provided unpaid care to an adult age 50 and over. Approximately 15.7 million family caregivers provided care for someone with Alzheimer’s disease or another dementia, totaling $600 billion in 2021 [22]. Such family caregiving constitutes the bulk of long-term care for older adults living in the community and may last many years, making the caregiving relationship a health policy priority.

Extant literature suggests older adults with comorbidities have preferences and priorities regarding their health and overall care [2, 7, 13, 27]. These may include priorities regarding access, modality of healthcare appointment, communication, or characteristics of providers that they find welcoming. Older patients and their family caregivers may hold incongruent perspectives about health and care that may compromise the caregiving relationship [1]. Incongruence documented thus far focused on the extent of patient’s difficulties, value of social relationships [17], autonomy [16], and preferred sources of socioemotional support [24]. Incongruence within dyads has been associated with relationship strain and caregiver burden [12]; these predict, in part, family caregivers’ desire to seek institutional care (i.e., seek alternatives to family caregiving) [26]. Supporting family caregivers of community-dwelling older adults may depend on recognizing and addressing incongruent perceptions within caregiving dyads.

Medical appointments including patients and family caregivers represent a setting that is well-suited for recognizing and addressing incongruent perceptions. Research on appointments attended by the patient, the family caregiver(s), and clinicians align with the positive effects patient-clinician interactions may have on patients’ emotional and physical well-being [25]. Yet we know little about patient and caregiver priorities raised during medical visits, the incongruence of these priorities, and clinicians’ responses to potential incongruence, especially in visits with older patients with varying levels of cognitive impairment. Our exploratory, qualitative study thus examines the types of priorities that patients with and without cognitive impairment and their caregivers express, any divergence of these priorities, and clinician responses.

### Conceptual framework

**Fig. 1:**
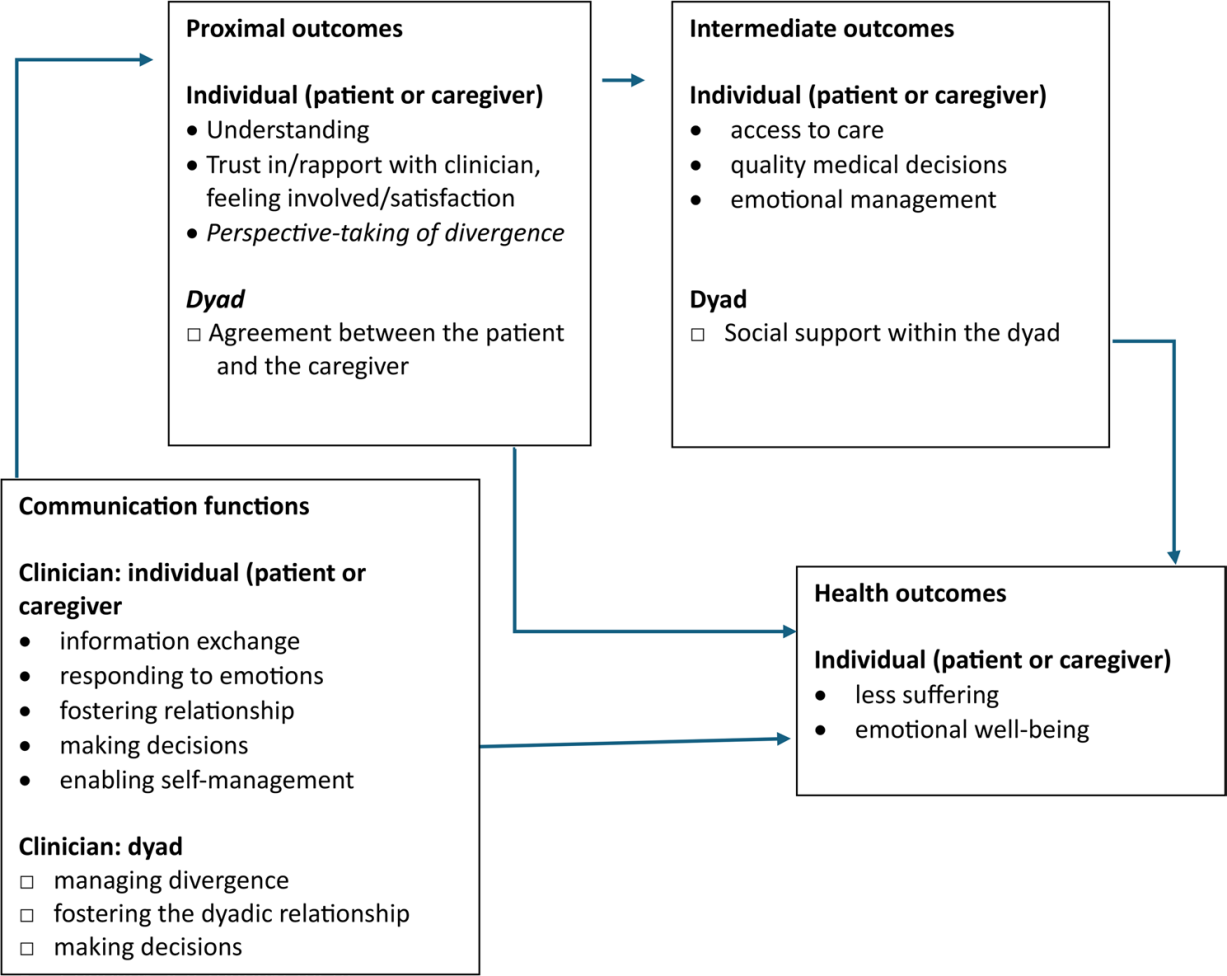
Our study’s conceptual framework of communication among the clinician, patient, and caregiver during medical visits, adapted from Street’s model of patient-clinician communication [25] Note: Our adaptations are preceded by □. For parsimony we omitted some individual intermediate and health outcomes from the original model [25]

Our study’s framework borrows elements from Street’s conceptual model on patient-clinician communication and health outcomes [25], which, in our study, focuses on communication among three parties, rather than two. According to this framework, communication during medical appointments with various parties may achieve different functions, such as information exchange, responding to emotions, facilitation, fostering relationships, and shared decision-making. While these functions may be accomplished by communication between the clinician and the patient (or clinician and the caregiver), some may be unique to communication between the clinician and the dyad (caregiver and patient). Such communication functions are not well-understood, including when there are multiple clinicians in the same encounter. See Fig. 1 for our study’s framework, an adaptation of the Street’s conceptual model of medical visits, incorporating communication functions involving dyads and dyadic outcomes.

## Methods

### Study setting and participant recruitment

Our study was conducted at [Blinded for Review] VA hospital’s outpatient geriatrics specialty clinic. The study’s purpose was to assess how clinicians navigate discussions with patients and their caregivers about their priorities, i.e., “what matters most” (WMM). The clinic consists of a geriatrician, nurse, social worker, and clinical pharmacist, with administrative support from a medical support assistant. Typically, first-time appointments are jointly attended by all available clinicians and, given VA’s priority to support family caregivers, include an extensive discussion about priorities of both the patient and the family caregiver(s). We therefore aimed to enroll patients attending their first-time appointments with a family caregiver.

Between July and September 2022, we approached all eligible patients (*n* = 4). During a phone call by the medical support assistant to confirm the appointment with the patient and family caregiver, one of the authors informed the patient or the family caregiver of the purpose of the study and invited them to participate in this study (i.e., have their upcoming appointment audio-recorded). All patients and family caregivers (*n* = 9) who were contacted during the calls gave verbal permission. Upon their arrival at the clinic, one author [Blinded for review] gave the patient and family caregiver(s) a written description of the study and asked for verbal confirmation that they agreed to have the appointment audio-recorded. After the patient and the family member(s) gave verbal assent, the audio-recorder was turned on and placed on the conference table, around which clinicians, patient, and family caregiver(s) sat.

Table 1 shows select characteristics of the four first-time medical appointments and the patients, caregivers, and clinicians who attended these appointments. These appointments lasted, on average, two hours. All patients were white male Veterans. Two of the four patients were diagnosed with dementia at the time of the appointments. Each appointment was attended by at least one family caregiver (i.e., spouse, child, sibling of the Veteran). All four appointments were attended by the clinic’s geriatrician, social worker, and clinical pharmacist, with one appointment also attended by the newly hired nurse.

**Table 1:** Select characteristics of four initial medical appointments, their patients, caregivers, and clinicians

	Patient 1	Patient 2	Patient 3	Patient 4
Appointment				
Duration (hours)	1 h:20 m	1 h:50 m	2 h:35 m	2 h:00 m
Patient				
Patient’s gender	Male	Male	Male	Male
Patient’s age	76	92	87	87
Race	White	White	White	White
Dementia diagnosis	No	No	Yes	Yes
Caregiver(s)				
# of caregivers present	1	1	2	1
Relationship of caregiver(s) to patient	Wife	Daughter	Wife & Daughter	Brother
Caregiver considered “primary caregiver”	Wife	Daughter	Daughter	Daughter
Clinicians				
# of Clinicians	3	3	4	3
Type of Clinicians	MD SW PharmD	MD SW PharmD	MD SW PharmD Registered Nurse	MD SW PharmD

The geriatrician, social worker, and pharmacist were the same clinicians in each appointment

*MD* Geriatrician, *SW* Social worker, *PharmD* Pharmacist

### Data collection and analysis

During the appointments, [Blinded for review] sat at a separate table in the same conference room. The physical positioning was designed to enable [Blinded for review] to listen to the conversation as unobtrusively as possible as well as observe and take notes of paraverbal (e.g., tone) and non-verbal communication. The audio recorder was turned off by [Blinded for review] at the end of the appointment (when individuals in the appointment stood up to prepare to leave the room). Audio files were transcribed verbatim by a professional transcriber. Transcripts were not returned to participants for comment or correction. Transcripts and field notes of paraverbal and non-verbal communication were uploaded to NVivo 12 [15] for code management and thematic analysis.

We performed our coding and analyses iteratively, guided by both an inductive approach rooted in the data [4, 5, 23] as well as by our conceptual framework. First, three authors ([Blinded for review]) constituted the coding team, each member independently listening to the audio recordings, reading the transcripts and engaging in, open coding of data elements (i.e., attach meanings or codes to excerpts of text). In group discussions, these three authors ([Blinded for review]) compiled notes and developed a first draft of the codebook of codes and their definitions. We used the codebook and coded in pairs ([Blinded for review]) to promote consistent coding. In follow-up meetings between coding pairs, coding discrepancies were discussed and resolved, and codes and their definitions were modified, with all changes made by coding pairs subsequently discussed and agreed upon in meetings with the entire coding team. We also sought feedback during the coding process from our fourth co-author to promote reflexivity and methodologic rigor. Our coding produced 24 codes that captured key concepts from the medical appointments. Our current study focuses on 22 codes describing patient and family caregiver’s priorities and the clinician’s responses. All authors ([Blinded for review]) then contributed to data interpretation.

We then performed thematic analyses from our codes [4, 5, 23], analyses rooted in a realist theoretical position to report on participants’ experiences [4] and informed by elements of the Street’s conceptual framework. To facilitate development of themes, we grouped codes into categories by speaker (i.e., patient-, caregiver-, clinician- codes). We grouped priorities by source (i.e., patient’s or caregiver’s priorities) and clinician responses by type (e.g., validation and acknowledgment). During thematic development, we also examined the content of our codes against elements of Street’s model, noting when codes were not explicitly specified in the Street’s model. Our analysis was performed by a set of coders consisting of researchers (PhDs in gerontology and education, with formal training in qualitative research methods) and a team member (PhD in clinical psychology with neuropsychology focus) who provided clinical care. In brief, to enhance rigor and consistency, we sought feedback from each co-author throughout the thematic development and data interpretation, including group discussions and resolutions of coding discrepancies. See Appendix Table 3 for our codebook of codes and their definitions. Member checking was not included. In other words, we did not return to the participants to present our data or results for their feedback or validation.

### Ethical considerations

This study was approved by the Research and Development Committee of a Veterans Affairs Medical Center in Veterans Integrated Service Network 1 [exact name of institution omitted during blinded review and in publication] as quality improvement to examine and improve clinician-patient-caregiver communication during WMM medical appointments. Our study was therefore not subject to Institutional Review Board oversight, and the need for informed consent to participate was deemed unnecessary according to the US Department of Veterans Affairs Office of Research Development Program Guide 1200.21(22). Nevertheless, we adhered to ethical principles throughout our study (e.g., all participants in the study were provided with a written document describing the voluntary nature of the study; verbal agreement to participate was obtained from all participants in the study prior to study participation).

## Results

To illustrate our themes and codes in context, we selected three scenarios. In each scenario, we first describe the viewpoints of the patient and the family caregiver and the extent to which they diverge, whether disagreements were explicit or more nuanced (implicit), and subsequently describe responses by clinicians. Within each scenario, we annotate selected passages (using brackets and bold text) that exemplify the clinical responses to divergence underlying our themes. In addition, to the extent possible without video recording, we annotated observations of para-verbal and non-verbal communication (in brackets and italicized text) to provide additional context.

### Scenario 1: clinician responses to concerns regarding cannabis use to cope with Post-Traumatic Stress Disorder (PTSD)

A male Veteran (Patient 1) who attended the appointment with his wife described his struggle to find effective ways to cope with PTSD following his military service in Vietnam. Although both the patient and his wife agreed on the patient’s need to manage PTSD symptoms, they stated their own perspectives regarding the patient’s cannabis use as a coping strategy. Specifically, the patient strongly appreciated cannabis as a way to control his PTSD symptoms whereas his wife expressed her discomfort surrounding the source of cannabis and her lack of knowledge regarding choosing products with the “right” potency. In addition, the spouses disagreed on smoking in the house, as the patient stated, “If I could smoke it in my room and jump into bed, that would be okay. But that’s not going to work [for my wife]. Both parties, nevertheless, expressed their mutual desire to reduce cannabis’ side effects, such as the patient’s reduced mobility while under the influence. The quotes below show clinician strategies include acknowledgement and validation of priorities of each member of the dyad and reframing of the problem and pointing out common ground or mutual understanding between the spouses.

Wife: …I came home one time in the middle of winter and a really cold night, he was out on the breezeway, and I can’t get him up. I can’t get him -- I tried to get him up but can’t. …[*tone of concern*,* distress*].

Pharmacist: Sure. Number one that sounds like a really difficult situation for both of you [**clinician validates caregiver and patient**] and so, I just want to say that… it sounds like, [wife’s name], some of what you’re worried about …is safety [**reframes the problem expressed by caregiver and patient**]. Is him being safe, and I think that’s really valid…worrying about his safety, especially if you’re not there. And he’s [the patient] impaired… [Addressing patient] …what worries you…?

Patient: The same thing.

Pharmacist: Okay. Tell me a little more in your own words about that?

Patient: Well, I need something to control my -- the aftereffects of the flashbacks. The marijuana helps. But, like alcohol would have a devastating effect on me.

…

Pharmacist: …-- managing the symptoms of your PTSD is certainly something that matters to you. Your safety and feeling like you’re able to move around and do your day-to-day in light of having those symptoms controlled also matters to both of you. [**clinician reframes problem expressed by the patient; pointing out common ground**] It sounded like you told me, and I’d like to hear more, that the pain clinic has been very helpful to you [**pivots to care planning**].

In addition to pointing out common ground, the clinicians also calmly listened to divergent perspectives and, at times, frustrations about daily routines. For example, the caregiver expressed her frustrations about their differences in preferred meal and sleep routines, “then if I fix a supper, most of the time he won’t eat it. Or he’ll eat a third of it or something. … But then I say you got to stay up, don’t -- try not to doze and stay up and then maybe you’ll be able to sleep through the night. … [drove] me crazy,” even though the patient remarked plainly “I don’t have an appetite…I can’t eat”. As shown below, in the absence of a clear common ground, clinicians acknowledged the divergence and, in due time, shifted the conversation to care planning inclusive of proposed coordination with the dental office and nutritionist—agreed by both parties—to address the patient’s poor appetite.

Wife:. I don’t know what to do to get him to kind of more schedule to his life….

Pharmacist: Okay. So, it sounds like two different philosophies for sure just for day-to-day, both valid. I totally understand both sides. [**clinician’s acknowledgement of incongruence**; *calm tone*] ….

Wife: …I wondered …you’re [Patient] getting [cannabis] from a girl who grows it up in Maine, and … Dr. XXX said that some are different strengths, and some are stronger than others and you don’t know and…the pot store…talks about chronic pain…[and] relaxation… I don’t know enough.

Patient: You need to understand, and you never will, the horror that goes through my mind when I relive some of those events and those flashbacks, and then you’ll understand and none of you ever will.

Pharmacist: You’re right. You’re right. [clinician’s acknowledgement of patient’s priority]

Social worker: Agree.

Wife: So, what you really are saying is that you need something strong enough that it completely just zips your mind to nothing.

Patient: No, I’m not. I’m saying something about halfway there.

Wife: Okay.

Geriatrician: That’s helpful for us. … but there are … experts in CBD and other formulations of marijuana for pain. [**clinician pivots to care planning**] I’m excited to work with you to see what’s out there… what’s going to keep your mind at ease as far as his safety [**clinician’s acknowledgement of caregiver priority**] …is this helping me be able to do the things I love without making me feel medicated. Or what is that works for [the patient]. [**clinician’s acknowledgement of patient’s priority**]

Patient: Yeah. … I can’t be medicated like what she is describing. That can’t work.

Pharmacist: Okay. And I think we all agree on that one too. …

### Scenario 2: clinician responses to divergence on utility of doing Chair-rise exercise at home

A 92-year-old male Veteran (Patient 2) attended the appointment with his daughter, who expressed that her father’s engagement in at-home exercise was her priority. In response, the geriatrician demonstrated a chair-rise exercise to the patient during the exam portion of the appointment. Later in the appointment, the patient expressed that he did not wish to engage in the chair-rise exercise.

Geriatrician: So this particular sheet shows something called the chair-rise exercise. I know you mentioned that you have family members who are fabulous at physical therapy and physical education [**Clinician acknowledges caregiver priority**]. I wonder if you would be up for…a little bit of exercise… into your week? ….

Patient: Eh, I’ve done them before. I don’t want to bother with it.

Geriatrician : I gotcha. So, it doesn’t sound like it’s something that you’re eager to build into your busy schedule at this moment, but we’ll keep it for you in the binder and we can even talk about it a little bit more next time perhaps….[**Clinician states their priorities**, *non-judgmental/neutral tone*].

Patient: Okay.

Daughter: …[my aunt] likes to do her exercises…She’ll be 100 in January.

Pharmacist : Wow, amazing. [**Clinician acknowledgement of caregiver priority**]

Daughter: …Yeah, [my aunt] just feels it’s so important to be able to get up out of her chair, use it or lose it.

Pharmacist: Yeah, great. That’s good advice.

Daughter: Yeah, lose it or lose it. Yeah, she’s amazing.

Geriatrician : Certainly sounds like you have folks in addition to you being so active…so that’s wonderful. So I’ll keep this one in the binder for you.

In response to the emergent implicit divergence between priorities, the clinicians validated the patient’s perspective while echoing the caregiver’s view of the importance of exercise. The geriatrician utilized the opportunity to encourage the patient to re-consider physical exercise at a later time.

Upon the patient’s rejection of chair-rise exercise, the geriatrician then pivoted to the physical therapy and balance/gait training as an alternative (to chair-rise exercise) to improve physical activity and safety. The geriatrician also reframed the chair-rise exercise not as exercise but as falls prevention and safety, a goal for discussion in a future appointment, stating, “But in general we also just were so impressed…with your family…at that point we might like to talk more about falls prevention, always making sure that you’re safe.”

Both the patient and the caregiver subsequently agreed to these referrals.

Geriatrician: … if you would like to meet with one of our physical therapists for some balance or gait training, sometimes they will recommend an assistive device, whether it be a cane or a walker…. [**Clinician pivots to another care option**] We certainly could consider that.

Patient: Yeah, I’d consider it.

Daughter: And it’s only going to be a few minutes when they come…not a big investment on your part but it’s worth it.

Patient: Yeah.

Geriatrician : So…we’ll put in for the home safety assessment and…an appointment with the physical therapist… [**Clinician proposes health care for common priority**].

Daughter: Yeah.

### Scenario 3: clinician responses to divergent responses related to patient’s driving cessation

A 87-year-old male Veteran living with dementia (Patient 3) attended the appointment with his wife and daughter. Several times during the appointment, the patient noted regret and disappointment at no longer being able to drive (“I’ll never get used to [not driving] I look out the window and see if my car’s there. I know it’s not”). His wife quickly responded, with a polite smile, “Stop focusing on the car, move on.” To manage the explicit divergent responses to the patient’s driving cessation, clinicians used validation, pivoting to other priorities, such as the importance of getting to medical appointments and the family’s vacation spot, and then proposed the use of a transportation service.

Pharmacist: You have been driving since you were ten -- right?

Daughter: That’s right [laughing] yeah. ….

Wife: Yes, he has.

Geriatrician: … It’s very difficult. It takes a while for you to at least… wrap your head around that oh I’m not gonna drive anymore…[**clinician’s validation of priority**; *tone of empathy*] but we wanna make sure that all your goals are being met regarding things that matter the most to you, like … going to your doctor’s appointments, going to [vacation home]. So …we spoke with to your sister last time and we talked to her about resources…transportation [resources] [**clinician pivots to care option**]. So, I was wondering if she had a chance to look at it?

Daughter: Yeah, so mom arranged once so far to get transportation through the Council on Aging.

Wife: Um, yeah.

…

Geriatrician : But I – I definitely hear what you’re [patient] saying, the fact that it is so different from, you know, how you have lived up until this point. Always being able to jump into the car and it does sound like this is an incredibly important thing to you to still be able to do all the things that you could. [**validation**, *tone of empathy*] So, I think that for our team that will be a big focus because it’s so important to you. So, I really am very hopeful that the DAV [Disabled American Veterans] might be a good opportunity to be able to come back for the monthly farmer’s market and the food bank. [**clinician pivots to another care option**] … [we] will start to … troubleshoot together about other ways to get you to the things that you enjoy doing [**clinician reframes problem**]. You know, it really is so difficult to have something that you used to look forward to but you can’t – you feel like you can’t do anymore, so we definitely wanna be as supportive as we can with that. That also brings me back to the point of [reducing] so many medical appointments…[so you can go to your vacation home].[**clinician pivots to another priority**]….

Specifically, the clinician re-stated and validated the patient’s priorities, and even suggested that modification to the number of appointments could be made to prioritize the patient’s value of returning to his home vacation. In addition, the clinicians then reframed and pivoted to other priorities the patient had expressed, such as getting to his medical appointments and receiving food truck service.

### Cross-Scenario observations of clinicians’ non-verbal or para-verbal responses to divergence in perspectives

Clinicians also used non-verbal communication techniques when incongruence emerged between the patient and the caregiver. Specifically, they used such strategies consistently across medical visits and within visits, including in the three scenarios described above as the patient and the caregiver talked about their unique perspectives. Clinicians exhibited active listening such as making eye contact to exhibit interest and attentiveness, nodding to indicate empathy or understanding, and listening without interruption. Clinicians also exhibited warmth and humor, by engaging in casual conversation topics when they were raised by the patient or caregiver (e.g., chatting about an upcoming sport event that the patient was attending, and laughing at jokes made by the patient). When incongruence emerged between the patient and the caregiver, or when the patient did not agree with the clinician’s recommendation, as with the chair-rise exercise, clinicians continued to speak with warmth and calmness and without judgement. Without taking sides, clinicians still validated each party’s priority to carry the conversation forward.

Taken together, the clinician responses align with concepts of communication functions in medical visits [25]. Table 2 displays a crosswalk mapping these communication functions (responding to emotions, fostering relationships, managing uncertainty/divergence, information exchange, making decisions, and enabling self-management) with our codes related to clinician responses and themes. For example, the communication functions of responding to each party’s emotions and fostering the relationship are achieved by provider acknowledgement of patient or caregiver priorities or addressing socioemotional needs of each party. In this way, the clinician navigated divergence while allowing all parties to feel heard and fostering a sense that all parties were working toward a mutual goal of care.

**Table 2:** Crosswalk of Codes, Themes, and conceptual framework’s communication functions

Code(s) Related to Clinical Responses/Skills Involved	Themes Identified	Communication functions
Clinician acknowledgment of caregiver priorities Clinician acknowledgment of patient’s priorities Clinician’s acknowledgement of differences between caregiver and patient priorities (acknowledgment of incongruence)	Addressing socioemotional needs	Responding to emotions Fostering relationships
Clinician reframes problem expressed by either the patient or caregiver Clinician pivots to another priority or care option	Seeking common ground	Managing uncertainty/divergence
Clinician gives information about health care Clinician proposes health care for common priority	Proposal to care planning	Information exchange Making decisions Enabling self-management

## Discussion

Our study characterized older adults’ and family caregivers’ divergence in health and lifestyle priorities expressed by older adults and their family caregivers. The study also identified clinicians’ responses when managing divergence in priorities during medical visits. We explored the content of the communication because the functions carried out during medical visits (e.g., information exchange) (a) may have important health outcomes on patients and their family caregivers as noted by [25] and (b) presents a unique opportunity and setting to explore divergence of priorities, and (c) to provide clinicians with guidance for navigating these encounters.

During these exchanges, explicit and implicit incongruence emerged. Specifically, older adults and their family caregivers often did not explicitly state that they disagreed with one another, which may indicate their preference to avoid confrontation. Family caregivers may find it difficult or uncomfortable to openly disagree with patients during medical appointments, as they strive to balance their own self-expression with their support of patient involvement [6]. Patients may not express disagreement with their family caregivers during the medical visit due to potential difficulties comprehending discussed information [10] or have difficulty finding the right words to express what they want to say, if a certain level of cognitive impairment is present. Nonetheless, patients may feel ostracized, isolated, or inferior [19] when their family members and clinicians focus on and discuss their “weaknesses” in such detail. Patients’ behavior may, in turn, inadvertently spur family members to take a leading role in the medical visit [30] and therefore voice their own priorities more. This is consistent with differences between patients and caregivers’ levels of engagement and contributions during the medical visits we observed.

### Clinical applications

Given these delicate and complex dynamics during medical visits, our analysis supports clinical strategies that foster candor and promote “balanced” involvement from both the patient and the family caregiver. Clinicians need to exhibit an openness to hearing and exploring divergent views [25]. Specifically, they need to convey (through verbal and non-verbal communication) to the patient and the family caregiver that both agreements and disagreements are normal and benefit from compromise [10]. If they do not, divergent views that may already be a source of relational strain can add to caregiver burden and patient’s own difficulty managing symptoms [25]. To demonstrate openness and promote perspective-taking by each dyad’s member during visits, clinicians could directly solicit patients’ thoughts on caregiver-expressed priorities and vice-versa. Another strategy to foster candor includes holding separate conversations with the patient and with the caregiver(s), prior to a joint meeting with both parties [8, 9, 20, 28]. This would allow patients and caregivers to voice priorities separately and subsequently come together to solve problems and decide on the next steps. This strategy is especially valuable for patients with cognitive impairment because, in separate conversations, clinicians could tailor and simplify information to help patients understand, encode, and retain it. Given that such communication skills take practice and education, clinicians may need training that includes role-playing related to disagreements (e.g., “act or verbalize your response to divergent views from various caregivers and patient surrounding driving”) and negotiation training [18]. Such training may be delivered via clinical consultation groups, webinars (which VA provides and continues to develop), and structured peer support. Such training could be instrumental in educating clinicians and pioneering further work in this area [21].

Several evidence-based clinical strategies observed in our study may be adopted by other clinicians. Validation and reframing are consistent with effective communication strategies used in health care [29], accompanied by elements of warmth and humor. Although not necessarily targeting disagreement, conveying warmth (smiling), calmness, and humor have been previously shown to benefit patients [11]. Patients and family caregivers also appeared comforted by the team’s counseling and care coordination, consistent with prior research [11, 13, 21], as such care coordination facilitates access to care. One way to strengthen effective communication is to form a clinical consultation group that targets disagreements during visits. Such a group could raise awareness of other difficult situations during medical visits with patients and their family caregivers. An example of one such difficult situation is of clinicians supporting caregiver-expressed priorities, which may build alliance with the caregiver but compromise rapport-building with the patient. A clinical consultation group could also be a forum for clinicians to discuss their own priorities during medical visits and how these priorities may influence the dynamics between patient and the caregiver and ultimately the outcome of the visit [3]. As such, these complexities during medical visits with patients and caregivers necessitate creative communication approaches and further study.

### Study limitations and research implications

The strengths of the current exploration involve the inclusion of real-time conversations during medical visits with dyads or triads of patients, at least one family member, and multiple clinicians. Analysis of these conversations brought to light types of disagreements among the three parties and clinician strategies employed to carry out conversations on WMM. However, our study also had several limitations that can inform future research. At the time of this writing, we were unable to ascertain the level of cognitive impairment of two patients in our study due to lack of cognitive screening and neuropsychological evaluation performance. We therefore were unable to address how levels of cognitive status may affect the expression of priorities (e.g., frequency and types) in medical visits, though current literature in this area does indicate that cognitive status and expression of priorities via engagement in medical appointments are related [14]. Future work on patient-caregiver-clinician communication should measure cognition specifically and comprehensively, as cognition can be viewed as being on a continuum, spanning from no cognitive concerns to subjective cognitive decline to mild cognitive impairment to dementia. Another limitation pertains to the nature of appointments in our study setting. Within Veterans Affairs geriatrics clinics, medical visits may be concurrently attended by members of an interprofessional team, so that the variation and nature of provider responses may have been directly influenced by the presence of multiple professions, hindering generalizability of findings to medical visits attended by a single clinician. The initial visits that were the focus of the current paper were also lengthier (2 h = mean duration) than typical general medical visits that older adult civilians or VA patients may receive (i.e., 20–45-minute visits). Furthermore, while there was a research observer in these medical appointments taking field notes, we did not conduct a systematic analysis of non-verbal and para-verbal communication because these appointments were not video recorded. Further, our sample was not representative of different cultural backgrounds. Since extensive literature indicates that culture influences perceptions of mentation (cognition and mental health) [19] and thus may influence report of priorities, future research should investigate how cultural background, and sociodemographic factors influence “what matters most” conversations and navigation around divergence in priorities. Such research will inform the development of practice guidance for “what matters most” conversations in underserved populations and prepare health systems to deliver age-friendly care in a culturally informed manner. In addition to overcoming these study limitations, future studies should examine how longitudinal changes in types of and self-reported ratings of priorities influence patient and caregiver proximal and intermediate outcomes.

### Summary/Conclusions

Our exploratory study focused on divergence in health and lifestyle priorities of patients with and without cognitive impairment and their caregivers and identified themes of clinicians’ responses in navigating such divergence. Although our study does not investigate distal outcomes, our study elucidates clinicians’ responses (a mix of addressing socioemotional needs, finding common ground, and care coordination to address commonly-held priorities) that likely foster proximal (perspective-taking by the patient and the caregiver) and intermediate outcomes (strengthen social support or mutual understanding within the dyad, access to care). In light of the exploratory nature of our findings, more research is still needed to understand divergence and clinical responses using larger and more ethnically and cognitively diverse samples of patients in various clinical settings.

## Data Availability

Data availability is contingent upon completing the requisite ethical requirements. Contact corresponding author for information regarding these requirements.

## Appendix 1

**Table 3:** Codebook generated from the qualitative analysis of the study’s initial medical appointments

Name	Description
***Caregiver behaviours***	
CG agrees with provider, accepts provider’s proposed next steps	Caregiver verbalizes a yes/okay response to provider’s next steps (e.g., safety checklist, home safety assessment, physical therapy referral, GeroFit/at-home physical therapy, getting a physical)
Caregiver asks patient a question	Caregiver asks patient a question/restates the provider’s question Ex: “Should we talk about that at home a while?” “I don’t know if it’s – You still have trouble getting words out”; “How often do you feel like you lack companionship?”
CG asks providers about health care	Caregiver asks provider about health care (code 1 question as 1 or multiple questions about the same concern, i.e., why multiple primary care docs, 1 VA and 1 Community) (such as option to contact providers directly when they have a question about the Veteran, about duplicate labs and duplicate primary care doctors, whether multiple family members can attend nutrition consult, etc.)
CG disagrees/rejects provider’s proposed next steps	Caregiver rejects a provider’s proposed solution (e.g., transportation, geri-psychology services, low-vision medication labels/medication scanner)
CG states priorities, concerns, and preferences	Lifestyle (diet, exercise, social/family activities, 2. Medical care, symptoms, must be stated by the caregiver and not the patient (e.g., hydration, medications, keeping parents at home, diet, memory, mobility, getting out and about, fall prevention, getting all family caregivers on the same page about Veteran diet, same-day transportation services) Priorities can be for self, patient, or other CGs.
***Group behaviours***	
Group debate and discussion about a specific course of action or issue	At least 2 people in the appointment openly debate and argue with each other/Confronting differences/No decisions necessarily made/providers could debate amongst themselves, group problem-solving; group members informing each others Ex: how patient/family should cope now that he no longer drives, family caregiver going out shopping
Group Alliance	Participants create an alliance
***Patient behaviours***	
Patient specifies role of CG for visit	Patient wants to decide with the caregiver collaboratively, patient agrees for providers to ask CG about the issues/what is on their mind/Patient defers to CG Patient prefers to have caregiver voice their opinions/defers to their judgment (EX: “She’s the boss”) i.e., Patient says I leave it up to my wife/daughter and they will respond/decide in place of me
Patient agrees, accepts provider’s proposed next steps	Patient agrees with next steps (e.g., meet with PT)
Patient defers to their doctor	Patient leaves the question/decision up to their doctor
Patient asks provider questions	Patient asks providers questions
Patient rejects provider’s proposed next steps	Patient rejects next steps (e.g., says No thank you, “I don’t want to bother with it” [chair-rise exercise])
Patient states priorities, concerns, and preferences	Lifestyle (social, work, exercise, mobility, diet) or medical care/specific symptoms; must be stated by the patient (EX: agrees it is important to move around, family, VFW activities/helping people, “keep going”/maintain health, keep refereeing boxing, misses pastimes of socializing at the foodbank, how to get the rollator downstairs)
***Provider behaviours***	
Provider acknowledgement of CG priorities	Provider acknowledges and/or validates a CG priority, concern, or preference (EX: acknowledges concerns about falling, being mobile – “Sounds like it’s very important” “It’s hot right now so we understand” in response to “We try to keep him drinking but it’s a battle”)
Provider acknowledgement of patient priorities	Provider acknowledges and/or validates a PT priority, concern, or preference **We coded all occurrences of acknowledgements, regardless of topic/concern Ex: “we know that that is an adjustment. It is very, very difficult”; “Sounds like your health, your good health is very important.”; “But that is so difficult, you know.”
Provider acknowledgement of differences between CG and patient priorities	Provider verbalizes there are multiple priorities in conflict Ex: whether to up the medication dose, CG says to “get over it” in relation to the car/not driving and provider says, “that is so difficult, you know.”
Provider asks CG questions	Provider asks CG about how to make health care treatment “convenient”, “doable”? Ex: “So, I wonder what you think – what we all think kind at this point in time, what should our plan be with the medicine going forward?”
Provider asks patient questions	Provider asks patient about their healthcare Ex: “So, I’ll start with you, XXXX, any questions that you have for our team?”
Provider reframes problem expressed by patient	Provider reframes a problem as something that can be overcome Ex: patient is sad about not driving and provider reframes stating “you still can meet your goals” and “they help you go places”; provider reframes being sad about moving from ME to the positives of being around family in MA
Provider encourages patient to adopt behavioral change	Provider encourages patient to adopt a change: To include patient activation strategies (asking for pat opinion, asking for understanding, paraphrase and interpret what’s said by pat) (e.g., hydration, exercise) Ex: “it seems like you do a lot of walking. A lot of walking so you might be thirsty”; provider encourages patient to exercise at home like their 100-year-old aunt
Provider gives info about health care	Patient education Ex: provider describes safety checklist in the take-home binder; patient education about cognitive changes/memory loss and treatments; patient education about availability of nutrition consult to lose weight; gives information about VA lab work for primary care; patient education about health care proxy form
Provider plans to coordinate with other providers	Provider plans to coordinate with other providers, including proposing consultations/referrals; ** when we code this, we code one time per visit for coordination around a specific issue** (discussed on 5/12/23) EX: “Sounds like it’s of interest to you all probably and maybe we can get some more information as to given the situation with XXXX’s amazing support system, what makes sense from a nutrition team and then we can tell you next time maybe what the options would be or give you a call and then see if we can set something up that works for everyone.”/coordinate with primary care for ear wax removal
Provider proposes health care next steps	Provider proposes a plan of action related to health care and social needs, including proposing consultations/referrals Ex: Audiology consult, DAV and the foodbank, plans to discuss medications next time, schedule next appointment “And it’s sounding like we might want to talk about that more next time and um, sounding like XXXX and you both have a great relationship and sounding like the two of you both help each other and spend a lot of time together and if I have any questions about medicines or day-to-day stuff that between XXXX and XXXX might be the person to ask.”
Provider states their priorities	Provider states their priorities, including key parts of their job (lifestyle changes), can be direct or indirect Including proposing to delay or visit a priority at a later date. Ex: “It’s good for you guys to still be able to continue to get out and do your own shopping”; “it might be helpful for us to have bigger conversation around some of these elements next time and we can certainly put the form in for the healthcare proxy.”; “we spoke with your sister last time and we talked to her about resources in – there in Arlington – Arlington and the Council on Aging and senior services. They all provide transportation. So, I was wondering if she had a chance to look at it?”; “So important to be prepared. Have the resource available. So I’m glad you guys had a chance to look at it.” [talking about driving service]/hopes to “cluster visits and appointments”

## Appendix 2

**Table 4:** Example priorities of patients and family caregivers across four initial medical appointments

Priority and definition	Patients	Family caregivers
**Medication-specific priority**: Priority around medications being taken by the patient, to include adherence, review and reduction, side effects, and alternatives	**“**I have on occasion. Right now, I didn’t take one today and my pain level is about five. So, I’ll rush home and I’ll probably will have to take two when I get there. It’ll probably be up about seven.” (Patient 1)	**“**So, the omeprazole really isn’t covering it and now I haven’t been giving him that because he’s taking the sucralfate -- sucralfate which he is supposed to take before he eats but sometimes he doesn’t remember.” (Wife to Patient 1) “…I wanted to check in was to see if he needed all of those? [medications] Because I don’t think they’ve been looked, you know reviewed in quite a while.” (Daughter to Patient 2) “I’m thinking it’s [donepezil] not hurting if he doesn’t have side effects, he should go on with the 5 mg for another month.” (Wife to Patient 3)
**Mobility-specific priority**: Priority around patient balance, walking, exercising, or pain related to mobility	**[**falls prevention] “That’s the important thing” (Patient 2) “…I’m not fit to drive, and I’m not fit to take a walk outside unless someone joins me.” (Patient 4)	“Mobility, we definitely want to keep him walking and the balance issue. My brother, my youngest brother’s a phys-ed teacher so he comes and makes him to lunges and exercises and get down on the floor and do stuff … he’s very, very, very mobile. He likes moving through space. The whole family, but he likes to move so we know that and try to make sure that we get out and go do something every day. That’s um, yeah. That’s the big one.” (Daughter to Patient 2) “PT has been – I don’t know if PT is offered here but he – he’s going – that’s been a big goal this summer cause in addition to the Alzheimer’s diagnosis, he’s [the patient was]… in terrible pain [in] winter and spring…he desperately needed physical therapy.” (Daughter to Patient 3) “… he does ambulatory walking like that almost every day to keep him mobile and not sedentary…. I mean we’re – we’re trying to keep him active on the street we live by walking up and down with XXXX, his sister-in-law. … They’ve asked him if he wanted to go and he just – he doesn’t feel like he’s ready to walk with XXXX, what they do.” (Brother to Patient 4)
**Mentation-specific priority**: Priority about memory challenges or fears like getting lost, or management of PTSD or other mental health conditions		
**Mentation –** Dementia/Cognitive impairment	**None**	**“**We’re looking at…options…for improving or helping [the patient’s] memory.” (Daughter to Patient 2)
**Mentation-** Mental health (e.g., PTSD)	“the flashbacks are so, physically and emotionally draining and they’re so, discouraging. It’s like being back there. It’s like being back there over and over and over and over again. And it takes -- every one of those episodes takes a month off my life. I can feel it happening. … But that’s not the end of it. For the rest of the day my soul is drained.” (Patient 1) “No, not adjusting well [to the move] … I miss what I had and that’s all that overtakes my whole life. … I’ve done all that and it doesn’t belong to me anymore. … I’m very homesick.” (Patient 4)	**None**
**Mentation –** Drug induced changes (e.g., effects of cannabis or alcohol)	“I can’t drink alcohol so, what I’ve done on occasion is smoke cannabis and that helps -- it helps. [laughing] It’s so disabling to me that I can’t function…Well, I need something to control my -- the aftereffects of the flashbacks. The cannabis helps. But, like alcohol would have a devastating effect on me. If I could smoke it in my room and jump into bed, that would be okay. But that’s not going to work…So, what the hell do I do?” (Patient 1)	“I have come home and he’s been on the breezeway, can’t get in the door. He…can hardly walk. … I’m concerned… Is there is something that he could take that wouldn’t change him.? … if he has to, to do something that doesn’t affect him -- if it does help the PTSD but I can’t stand it when he can’t stand up and his whole face and he’s just -- so, medicated that, you know, he’s a danger to himself.” (Wife to Patient 1)
**Other health or healthcare priority**: Priorities raised including sleep, diet, hydration, dentition, dizziness or tremors, need for services like physical exams and checks of pacemakers, reducing number of appointments, home health, incontinence, and falls prevention	“I can’t eat. I don’t have an appetite.” (Patient 1) “I used to – look forward to having my pacemaker checked. It is a vital instrument if you wanna call it and it’s amazing we haven’t – I haven’t had – I haven’t gone to the hospital and had my pacemaker checked” (Patient 4)	“Also, he doesn’t eat that much. … Yeah, and then if I fix a supper, most of the time he won’t eat it. Or he’ll eat a third of it or something. … Then he watches TV until 3:00 or 4:00 in the morning. He goes to bed and then he gets up 9:00, 10:00, 11:00 whatever, when he wakes up. So, he’s not getting a real good night’s sleep. … he’s been having a lot of dental work. He doesn’t have many teeth that had issues. Dr. XXXX that has been doing it -- he’s been trying to get a hold of Dr. XXXX. He cannot -- he’s had several implants put in, but it seems like we haven’t heard from him in about six, seven, eight months. … I was wondering whether you people could push the dental to get his teeth done completely so, he can eat again. …. I would like you to have a -- sort of a physical here because you know how I feel about the -- your last visit.” (Wife to Patient 1) “We try to keep him drinking but it’s a battle. … we’d like to incorporate diet but it’s hard because they don’t like change and they have certain foods they like. … Do you ever do that [full nutrition appointment] with the family or could more family members come to that?” (Daughter to Patient 2) “… Like is there a way to coordinate this so there’s fewer doctor’s appointments, at least for him. … … he’s had spells of like dizziness – like near fainting.” (Daughter to Patient 3) “…as far as for the monitoring your pacemaker we – they can do that remotely once we get that set up for you, which we don’t have right now. That would – that would appeal to him for his peace of mind knowing that his pacemaker is in good shape, even though I have checked it. … My concern with [the patient] is to– make sure he’s getting the adequate care that he needs …we need to establish him with a cardiologist …” (Brother to Patient 4)
**Patient lifestyle priority**: Priorities to include living independently at home, sex, daily routines including social and physical activities, what matters most like family and having regular calls, past hobbies, driving, past living arrangements, being outdoors, and storage of exercise equipment, isolation and safety priorities.	“Sex is on the top of my list, but sleep is next.” (Patient 1) “.Just [stay] as healthy as I am and doing the things that I do and helping veterans whatever way that I can. … My family. It’s my family. They’re -- they’re with me, around me almost daily and different parts of the day. I have a couple of sons. … I was president for three or four years and we have a VFW club in XXXX that I have been president of for a long while. And I’ll do anything for the veterans association. I will try and help. … Yeah, it’s wonderful and satisfying for me. A lot of fellas have come home from the wars and are not quite themselves. And we work well with them. We work well with them and at the VA hospital too. … if I’m available to do a boxing referee I take into consideration who they are, whether they’re heavy weights or whether they’re not heavyweights and I don’t, I don’t put myself in any problems.” (Patient 2) “I look out the window and see if my car’s there. I know it’s not … Here’s another thing that I miss… Once a month, there was one of the girls that always come up to the cars and I miss it [the drive-up food bank].” (Patient 3) “…the one thing I miss more than anything is driving.” (Patient 4)	**“**And I make him walk the dog. [laughing] He goes out five to six times. I think to just walk him out to the yard and back. … I’m constantly on that he doesn’t have a schedule. He’s not scheduled enough and that’s why he’s having all these issues. He doesn’t get up and have a breakfast.” (Wife to Patient 1) **“**But our combined goal is to keep them home and together. So pretty much almost whatever we have to do we’re all willing to do that. Right now, his wife’s kids are at the house more often. I come down and stay one night a week, you know so two days. My brothers, we all take turns coming down. So that’s, that’s the goal. The overarching goal.” (Daughter to Patient 2) “believe me it was not easy for me to go up and tell him that we had to do this for his health [alter living arrangement] – but it’s better… we had to bring him with his family,, so – to be safe and to be – to show that we all love him and we’re gonna take care of him.… when you realize that and he does realize that it makes him say okay well, you know, it had to be, what had to be and we’re doing what we have to do to keep – continue on in a different chapter of life. And negatively speaking, he could’ve been in an assisted living facility and be alone and be – that’s not him… Now the good thing is, he’s with his family. You know, he’s with his 2 brothers who are there caring for him and he’s in a good home environment, in a nice home with a dog he pats all day long.” (Brother to Patient 4)
**Caregiver lifestyle priority**: Priorities that relate to the caregiver(s)’ own lifestyle including self-care, flexible transportation, social engagement, need for a break, feelings of overwhelm, and desire for siblings to get “on the same page.”	**None across all four appointments**	“I’m probably the one who needs this more than him. … this year it was very hard for me to decide whether to go [visit her sisters] or not because of leaving him…I worry about him… But I know when I got down to my sister’s, it was one of these -- she said what do you want to do. I don’t want to make any decisions for one week.” (Wife to Patient 1) “I mean, I’d love to have them bump up the antioxidants on a consistent basis and it’s hard. And I would like to have my brothers and everybody else. It would be nice if everybody got on the same page and was a little more aware of the nutrition. Because if I am, it doesn’t matter if everyone brings them donuts every day.” (Daughter to Patient 2)

*Notes. Priorities are defined as emergent needs expressed spontaneously or following clinician prompting, that reflected problems in medical care or lifestyle, or what is important in general (including hopes and aspirations). Preferences are approaches in treatment or lifestyle. For our coding, we grouped these categories into one code

## Abbreviations

CG: Caregiver
